# Validation of Self-Regulated Writing Strategies for Advanced EFL Learners in China: A Structural Equation Modeling Analysis

**DOI:** 10.3390/ejihpe13040059

**Published:** 2023-04-21

**Authors:** Xuan Wang, Jianting Ma, Ximeng Li, Xinyi Shen

**Affiliations:** 1 School of Foreign Studies, Northwestern Polytechnical University, Xi’an 710072, China; 2 Institute of Linguistics, Shanghai International Studies University, Shanghai 201620, China; 3 Centre for Multidisciplinary and Intercultural Inquiry, University College London, London WC1E 6BT, UK

**Keywords:** multidimensional model validation, self-regulated writing strategies, SEM, EFL learners

## Abstract

This study aims to validate self-regulated writing strategies for advanced EFL learners through a structural equation modeling analysis. Two sets of advanced, university-level EFL learners in China were recruited on the basis of results from a nationwide standardized English test. Sample 1 consisted of 214 advanced learners and served mainly as a data source for exploratory factor analysis. Sample 2 consisted of 303 advanced learners; data from this group were used to conduct confirmatory factor analyses. The results confirmed the goodness of fit of the hierarchical, multidimensional structure of self-regulated writing strategies. This hierarchic model has the higher order of self-regulation and the second order of nine self-regulated writing strategies that belong to four dimensions. In terms of model comparisons, the indices of Model 1 (nine-factor correlated model of EFL writing strategies for SRL) and Model 2 (four-factor second-order model of EFL writing strategies for SRL) mark significant improvements in terms of fit over the indices of Model 3 (one-factor second-order model of EFL writing strategies for SRL). This means the four-factor model (cognition, metacognition, social behavior, and motivational regulation) offered a better explanation for advanced EFL learners than the model treating self-regulated writing strategies as a single convergent factor. These findings, in some ways, differ from the results of earlier research on EFL learners’ self-regulated writing strategies, and the findings of this study have certain implications for L2 writing teaching and learning.

## 1. Introduction

Second-language (L2) writing is one of the most important language-learning skills that English as a foreign language (EFL) learners should acquire. It has gained wide attention in the past decade [1,2,3,4,5,6]. However, L2 writing is also one of the most challenging skills, as it is “recursive, strategic, and multidimensional” (p. 188) [7]. It also varies among groups of EFL learners and across a wide range of EFL contexts [8,9,10].

Self-regulated learning (SRL), a process in which learners manage their cognition, affects, and behavior to achieve their learning targets, plays an important role in the goal-oriented, multifaceted, cognitively demanding L2 writing production process [11,12,13]. Self-regulation is commonly acknowledged to play a crucial role in language learning; it contributes to promoting active, productive learning in specific L2 language skill areas and learning contexts [14,15]. Numerous empirical studies have explored SRL in specific L2 language skill and knowledge areas. Tseng et al. [16] pioneered self-regulation in L2 vocabulary learning and developed a corresponding questionnaire. Finkbeiner et al. [17] emphasized the cognitive and metacognitive aspects of SRL in L2 reading, while Huang [18] studied the motivational aspect of SRL in the context of EFL speaking. However, multidimensional studies on SRL in writing are relatively recent and scarce [19,20]. Studies of L2 writing within the SRL framework have focused exclusively on a single dimension (cognitive strategies or motivational regulation) because of the complex nature of L2 writing and the lack of an agreed-upon operational classification of L2 writing strategies [20,21,22].

SRL writing strategies have recently gained a great deal of attention in EFL contexts. Researchers have studied instrument development and validation [20,23]; instruments’ predictive effects on writing performance [24,25]; their relationship with self-efficacy and engagement [22,26]; and their influencing factors, including social factors and individual differences, such as gender, academic major, and grade level [27,28]. Instrument development is foundational for data collection when conducting empirical studies. Since Teng and Zhang [20] developed the Writing Strategies for Self-Regulated Learning Questionnaire (WSSRLQ), the first questionnaire in this field, various studies have validated the questionnaire before exploring further relationships or effects. The validity of the questionnaire has been widely explored in terms of educational level, including in EFL secondary schools and universities. However, the questionnaire’s validity for different language learners, which is based on language proficiency, has not yet been investigated. Advanced learners are an especially notable group because their metacognitive strategy usage frequency, flexibility, and control have shown different trends and features from those of other groups of learners at different language proficiency levels [29,30], which (metacognitively) is closely related to self-regulation and makes the validation of WSSRLQ for advanced learners worth investigating. This study aims to validate self-regulated writing strategies for advanced EFL learners. This could contribute to understanding whether a multidimensional model can explain advanced learners’ use of self-regulated writing strategies.

## 2. Literature Review

### 2.1. L2 Writing Strategies

L2 writing is a multifaceted, dynamic, context-based, challenging process [31] in which learners’ writing strategies play key roles in achieving their writing goals [32,33]. According to Manchón [34], writing strategies can be defined in both broad and narrow terms. Broadly speaking, writing strategies can be described as actions or behaviors involved in the writing process. More specifically, only certain writing actions and behaviors should be considered. The literature identifies four defining characteristics in this regard [35,36]: (1) strategies should be active (what learners do); (2) strategies should reflect a learners’ own choice; (3) strategies should have clear goals; and (4) strategies should be aimed at language learning. Writing strategies can also be described as the conscious actions, behaviors, or thoughts used for completing writing activities [37].

L2 writing strategies have been examined from linguistic, social, and cognitive perspectives. Studies from the linguistic perspective have focused on L2 learners’ mechanisms for writing different text types and genres [38,39,40] and their ability to transfer knowledge from L1 to L2 [41,42]. Studies from the social perspective have focused on how L2 learners cater to the discourse community’s social needs [43,44,45]. Studies from the cognitive perspective have focused on L2 learners’ mental actions during the writing of macroprocesses, including planning, formulation, revision, monitoring, and problem-solving [46,47]. Writing strategies from the cognitive perspective have received particular attention; the majority are based on self-regulation theories [22,24,26,48].

### 2.2. Self-Regulated Learning

Self-regulated learning can be described as a process in which learners actively participate in their learning [31,49,50]. Pinstrich [51] defines *SRL* as “an active, constructive process whereby learners set goals for their learning and then attempt to monitor, regulate and control their cognition, motivation, and behavior, guided and con-strained by their goals and the contextual features in the environment” (p. 453). SRL is a multidimensional construct comprising cognitive, affective, behavioral, and motivational aspects. During the learning process, learners’ cognition, affects, and motivation lead to active learning, enabling them to adjust their actions to different conditions. Through goal setting, monitoring, strategizing, and self-evaluation, learners effectively manage their learning and ultimately achieve their goals [16,52,53].

Current research on SRL can be divided into two main categories. One strand of research has explored the relationship between SRL and learning performance, while the other strand has focused on learners’ individual differences in SRL. SRL’s influence on academic performance has been extensively studied [54]. The empirical results show a significant and positive relationship between SRL and learning performance [55,56,57]. When learners engage more in SRL, their performance improves. SRL is important in guiding learners to monitor their learning and achieve effective learning outcomes in various contexts [53]. However, students’ ability to regulate their own learning differs [58]. Therefore, researchers have invested a great deal of effort in related studies and found that individual differences influence various aspects of SRL, such as motivations and goals [53], self-efficacy [59], and emotions [60].

To better explore learners’ cognition and regulation activities during the language-learning process, Dörnyei [61] first introduced SRL to L2 teaching and learning. He argued that learners’ SRL competence should be the focus and that research on language-learning strategies should be fully encompassed by SRL research. However, researchers have debated the relationship between language strategies and SRL in language learning. Whether the research should focus on strategies or competencies remains controversial. For instance, Oxford [62] argues that research on language-learning strategies and SRL research are intertwined and that both should coexist. Research on language learners’ writing strategies has been influenced by research on SRL in L2 [14]; driven by the abovementioned academic debate, more studies on how the different dimensions of SRL strategies influence writing have emerged in recent years [63,64,65]. Nevertheless, research on self-regulation in L2 writing is generally still new, and it shows great potential for future studies [15,35].

### 2.3. Self-Regulated Writing Strategies

Self-regulated learning in L2 writing has been defined as “deliberate, goal-directed attempts to make writing enjoyable, less challenging, and more effective” (p. 680) [20]. Even though research in the EFL context has only started being undertaken within the past 10 years, this area of study has experienced rapid development. The relevant studies can be divided into three main domains.

First, numerous studies have focused on developing and validating self-regulated writing strategy questionnaires for secondary-school students [23,28] and university-level students [20,21,22]. Teng and Zhang’s study [20] developed and validated a new instrument with a multidimensional structure based on SRL theory in order to understand and assess EFL learners’ self-regulated writing strategies. They evaluated three hypothesized models to provide a more defined perspective for self-regulating writing strategies. In the first hypothesized model, a first-order model was collectively formed by nine distinct but correlated factors of writing strategies for SRL. The nine factors were text processing, course memory, idea planning, goal-oriented monitoring and evaluation, peer learning, feedback handling, interest enhancement, motivational self-talk, and emotional control. In the second hypothesized model, a four-factor second-order model was proposed to examine whether the nine lower-order writing strategies for SRL could be conceptualized into four higher-order correlated factors, including cognition, metacognition, social behavior, and motivational regulation. Cognitive strategies involve skills for processing information in an assignment, while metacognitive strategies enable learners to control their cognition and apply their cognitive resources to complete tasks [66]. Social-behavioral strategies, which highlight feedback handling and peer learning, are designed to determine learners’ regulation of learning behavior influenced by teachers and peers. Motivational regulation strategies involve motivational skills that learners can use to willingly and consciously maintain their efforts in writing. In the third hypothesized model, a one-factor second-order model was proposed; a single common factor as a higher order, namely self-regulation, accounts for the correlations among the nine factors in the first hypothesized model. The results showed that all models had a favorable model fit, confirming the multidimensional model of self-regulation as a hierarchical construct with nine correlated factors that belong to the four subcategories of cognition, metacognition, social behavior, and motivational regulation. In terms of model comparisons, the third hypothesized model demonstrated the best model fit, in which self-regulated writing strategies were a higher-level construct with nine correlated factors that belong to the four subcategories of cognition, metacognition, social behavior, and motivational regulation.

Given the complexity of SRL, Teng and Huang [23] adopted the questionnaire-based multidimensional model developed by Teng and Zhang [20] to investigate the best structural model that represents self-regulated writing strategies in the context of secondary-school EFL learners. Teng and Huang’s results confirmed Zimmerman’s higher model, consisting of four dimensions: cognition, metacognition, social behavior, and motivational regulation. Moreover, as an integrated construct, self-regulation was found to influence EFL learners’ writing competence, as measured by a writing test modeled after the writing portion of the National Matriculation English Test. The nine writing strategies under the four dimensions of self-regulation were found to be closely correlated. Collectively, these findings aligned with Teng and Zhang’s results [20]; however, Teng and Huang [23] found that learners’ reported use of SRL strategies was affected by learners’ individual differences, which complemented Teng and Zhang’s study and helped explain differences between university-level and secondary-school students in using self-regulated writing strategies.

Second, the predictive effects of self-regulated writing strategies on writing performance have also been widely explored [20,23,24,67]. Teng and Zhang’s study [20] revealed some strategies, such as text processing in the cognitive dimension; idea planning and goal-oriented monitoring and evaluating strategies in the metacognitive dimension; and emotional control and motivational self-talk in the motivational regulation dimension, strongly affect learners’ writing outcomes. The authors’ study helped conceptualize SRL in the EFL writing aspect and, to some degree, establish pedagogical guidance. The results of Teng and Huang’s study [23] confirmed that nine self-regulated writing strategies had significant effects on secondary-school students’ writing proficiency. Sun and Wang [68] investigated the predictive effect of self-regulated writing strategies on Chinese college students’ writing proficiency by controlling for two influencing factors, namely gender and socioeconomic status. The findings indicated that self-regulated writing strategies can significantly predict writing performance after controlling for socioeconomic status. Teng et al. [28] reported the relationship between students’ use of self-regulated writing strategies and their writing performance in the context of EFL secondary school. Six strategies, namely writing plans, goal-oriented monitoring, goal-oriented evaluation, emotional control, memory, and metacognitive, were all significant predictors of secondary-school students’ writing proficiency. These findings suggested the importance of self-regulated writing strategies on adolescent L2 learners’ writing proficiency.

Third, the relationship between self-regulated writing strategies and individual differences has been explored. Results on L2 learners’ motivational domain, which includes interest, self-efficacy, and a growth mindset, were relatively consistent and showed that these motivational differences differently affected self-regulated writing strategies for learners at different competence levels [24,26,69,70]. However, studies on language proficiency and genders have been inconclusive. Qin and Zhang [27] and Teng, Wang, and Zhang [28] found significant differences among EFL learners at different language proficiency levels in China, while Mallahi [71] and Umamah, Khoiri, Widiati, and Wulyani [67] found that language proficiency levels did not significantly influence L2 learners’ self-regulated writing strategies in EFL contexts in Iran and Indonesia. Teng, Wang, and Zhang found that female learners used more self-regulatory writing strategies than male students did, while Shen and Bai [24] found that gender and academic major were not mediating factors that influenced L2 learners’ self-regulated writing strategies. In addition to individual variances, classroom environmental factors, such as the instruction approach for L2 learners (i.e., strategy-based instruction) and class engagement, could also influence or even improve learners’ self-regulated writing strategies [22,48,64].

Questionnaire validation has been the most prominent and compulsory investigation in the field of self-regulated writing strategies, and validation is foundational to collecting data when conducting empirical studies. The current study views the validation process as the collection of evidence with the goal of justifying the interpretation of the data and the subsequent use of the results. Since the development of the first questionnaire in the field, namely Teng and Zhang’s [20] Writing Strategies for Self-Regulated Learning Questionnaire (WSSRLQ), questionnaire validation has been widely explored in terms of education level, including at the secondary-school and university levels [21,22,23,24]. However, educational level may not be the only consideration for WSSRLQ validation. Language proficiency is another critical factor ripe for exploration: studies on the second domain (predictive effects of self-regulated writing strategies on writing performance) and the third domain (the relationship between self-regulated writing strategies and individual differences) have shown that language proficiency levels were the most researched variance, but the related findings were inconclusive. Specifically, advanced learners are a notable group because their metacognitive strategy usage frequency, flexibility, and control show different trends and features compared with less-proficient learners [29,30,72]. Such findings on metacognition are closely related to self-regulation and make the validation of WSSRLQ for advanced learners a vital aspect worthy of investigation. In order to address this issue, and to reconcile inconsistencies engendered by gaps in proficiency levels, this study aims to validate self-regulated writing strategies specifically for advanced EFL learners.

## 3. Methods

### 3.1. Participants and Research Settings

According to the Chinese Education Authority curriculum, secondary-school students should achieve intermediate-level English proficiency with a vocabulary size of around 3300 words, while university students should achieve upper-intermediate-level to advanced-level proficiency with a vocabulary size of around 6000 words [73,74]. This study aimed to validate self-regulated writing strategies for advanced learners; accordingly, the participants for this study were university students whose English proficiency level was assessed by both their latest total and latest sub-scores on the College English Test Band 6 (CET 6). CET 6, as a large-scale standardized test administered nationwide by the National College English Testing Committee in China, is a Chinese national standardized English test with high credibility that aims to measure undergraduates’ English proficiency at higher language levels [75,76,77]. The latest scores of participants refer to the time period when they participated in this study and filled out the questionnaire; these scores were achieved within 6 months prior this study to ensure that participants were exactly at the advanced level when participating in this study and describing their self-regulated writing strategies. The total score and the sub-scores were selected to ensure all participants were at an advanced level in both general language proficiency and writing. Specifically, participants who had both a final score over 438 points (total possible points = 710) and a writing and translation section score over 114 points (total possible points = 212) were recruited. These score lines (equivalent to the B2 level) were selected according to the latest mapping of CET 6 to the Common European Framework of Reference for Languages [78,79,80]. The CEFR B2 level has been commonly considered a starting point for advanced-level learners in both research articles [81,82,83,84] and university entrance requirements for overseas students (e.g., universities in the UK and Australia).

On the basis of the research aims and the advanced-level participant recruitment requirements, two sets of samples were recruited: (a) Sample 1 consisted of 214 samples, mainly collecting data for item and exploratory factor analysis (EFA), and (b) Sample 2 consisted of 303 samples used to conduct confirmatory factor analysis (CFA). In Sample 1, researchers sent out 319 questionnaires. Researchers then eliminated the questionnaires with obvious rule violations, those with missing answers, and those with substandard scores. In total, 214 questionnaires from five universities in northwest China remained, with an effective rate of 67.08%; 125 male (58.41%) and 89 female (41.59%) respondents, ranging from 18 to 23 years old, were included in the 214 questionnaires. In Sample 2, researchers sent out 439 questionnaires, and 303 effective questionnaires from five universities in northwest China were obtained, with an effective rate of 69.02%. In total, 162 male (53.47%) and 141 female (46.53%) respondents, ranging from 18 to 23 years old, were included in the 303 questionnaires. Further, 518 samples were collected, and no repetition or overlap of participants existed between Sample 1 and Sample 2. Both samples comprised more than 200 participants and thus met the sample-size requirements for structural equation modeling (SEM), for which the number of participants should be at least five times the number of items in the questionnaire (the WSSRLQ has 40 items) [85].

### 3.2. Data Analysis

Before constructing the self-regulated writing strategies model for higher-level EFL learners, researchers in this study first analyzed the general quality of the questionnaire. In the pre-stage, Sample 1 was used to conduct item and reliability analyses to verify the questionnaire item discrimination and dimension consistency. The critical ratio (CR) was used as a quantitative standard to measure question discrimination; Cronbach’s internal consistency coefficient was used to measure reliability. For the EFA analysis, this study used the 214 samples from Sample 1. First, the KMO values for 40 items were calculated, and Bartlett’s test of sphericity was used to determine whether the data were suitable for factor analysis. Next, principal component analysis and orthogonal rotation were used to determine the number of common factors, according to the Kaiser criterion and the Scree test of variance interpretation ratio. In the CFA stage, the 303 samples from Sample 2 were used, and the following three validity tests were conducted on the three models: the convergent validity test, the discriminant validity test, and the construct validity test. Finally, chi-square values were calculated to compare the three proposed models.

### 3.3. Writing Strategies for Self-Regulated Learning Questionnaire (WSSRLQ)

The current study adopted the Writing Strategies for Self-Regulated Learning Questionnaire (WSSRLQ), developed by L. S. Teng and Zhang [20], because of the extensive investigation of its construct validity for EFL learners at different educational levels. The WSSRLQ questionnaire contains 40 selective and synthetic items sorted into nine subcategories, which are text processing, course memory, idea planning, goal-oriented monitoring and evaluation, peer learning, feedback handling, interest enhancement, motivational self-talk, and emotional control. Those factors fall under four regulation dimensions: (a) text processing and course memory fall under cognition; (b) idea planning and goal-oriented monitoring and evaluation fall under metacognition; (c) peer learning and feedback handling fall under social behavior; and (d) interest enhancement, motivational self-talk, and emotional control fall under motivation. A 7-point Likert scale was chosen with gradation rating from 1 (not all true of me) to 7 (very true of me) to explore the trait features of self-regulated writing strategies. Even though WSSRLQ has been validated in various groups, this study sampled a previously unexplored group, advanced EFL learners. Therefore, this study first employed EFA to explore the WSSRLQ dimensions and then employed CFA to determine whether the proposed construct model was suitable for advanced EFL learners. This study also employed item and reliability analyses to test the reliability of the questionnaire and data before the EFA stage. The questionnaire used in this study is provided in Table 1.

## 4. Results

### 4.1. Item Analysis

In order to test the rationality of the WSSRLQ in this study, researchers conducted an item analysis on the discrimination and consistency of 40 items involved in the questionnaire. Critical ratio (CR) was regarded as the criterion for the discriminant of each item. Only items whose CR value reached a significant level were retained. Researchers separately calculated the total score of each questionnaire and took 27% as the critical point to distinguish between high and low scores. An independent sample *t*-test was conducted for each item to detect the significance of difference between the average scores of the high and low groups. The analysis results showed that each item in the questionnaire reached a significant difference level (−10.155 < T < −5.078), indicating that the questionnaire was able to discriminate between high scores and low scores. Meanwhile, the correlation between each item and the total score also reached the significant level of 0.05, and the correlation coefficient was higher than 0.3 (0.415 < r < 0.676), indicating the high internal consistency of each item in the questionnaire.

### 4.2. Reliability of the Questionnaire

Cronbach’s coefficient was considered to be the most commonly used index to evaluate the reliability of one questionnaire. According to Nunnally [86], a questionnaire with a coefficient of 0.7 or higher than 0.7 could be considered as a questionnaire with reliability. In this study, the coefficients of factors 1 to 9 of the WSSRLQ were 0.901, 0.797, 0.851, 0.879, 0.809, 0.865, 0.844, 0.906, and 0.822, respectively (Table 2). The coefficient of the whole questionnaire was 0.916, which was still greater than 0.7. Therefore, the WSSRLQ in this study had high homogeneity and reliability.

### 4.3. Exploratory Factor Analysis

The methods of principal component analysis (PCA) were used to perform the exploratory factor analysis on the test data of the self-regulated writing strategies of advanced EFL learners. Afterward, common factors were extracted from it, and the final factor load matrix was attained using the orthogonal rotation method. After several instances of exploration, we performed the second exploratory factor analysis on 40 questions without the low load (<0.4).

Through KMO and Bartlett’s test, KMO was 0.883 and the significance level of spherical test was 0.000, which shows that the variables are strongly correlated and that the data are suitable for factor analysis. On the basis of the criteria with characteristic roots >1, the research adopted SPSS 23.0 to perform a principal component analysis and the orthogonal rotation of the data, and nine factors were extracted. The explanation amount was 69.984%, with a factor load of 0.700. All these data indicate that the results of the factor analysis are ideal.

### 4.4. Confirmatory Factor Analysis

#### 4.4.1. Convergent Validity

Convergent validity refers to the aggregation degree of the observed variables corresponding to each latent variable and is generally evaluated by three indicators: standardized factor loadings, composite reliability (CR), and average variance extracted (AVE). According to Hair et al. [87] and Fornell and Larcker [88], if the factor loadings are above 0.5, CR is above 0.6, and AVE is above 0.5, the observed variables have convergent validity. As shown in Table 3, the factor loadings of each latent variable for each item are all over 0.7, which indicates that each latent variable is highly representative of the item it belongs to. Moreover, the AVE values of each latent variable are all over 0.5, and the composite reliability (CR) values are all over 0.8, which demonstrates that the convergent validity is satisfactory.

#### 4.4.2. Discriminant Validity

Discriminant validity refers to the low correlation and significant difference between latent variables. Fornell and Larcker [88] proposed that discriminant validity between two variables is present if the square root of the AVE for each variable is higher than the correlation coefficient between this variable and the other variable. Moreover, if the square root of the AVE is over 0.50, the latent variable has good reliability and validity.

The results of the test are shown in Table 4. The values on the diagonal in the table are the square roots of the AVE values of the latent variables, and the values on the nondiagonal are the correlation coefficients between the latent variables. It can be seen that the square roots of the AVE for each latent variable range from 0.752 to 0.803, while the correlation coefficients range from 0.043 to 0.501. The square roots of the AVE for each variable were significantly higher than the correlation coefficients, which indicates that the variables had good discriminant validity. Moreover, except between EC and PL, all other latent variables were significantly correlated with each other (*p* < 0.05), which suggests that these latent variables are somewhat correlated with each other but also discriminated; that is, the discriminant validity of the scale data is ideal.

#### 4.4.3. Construct Validity and Model Comparisons

**Figure 1:**
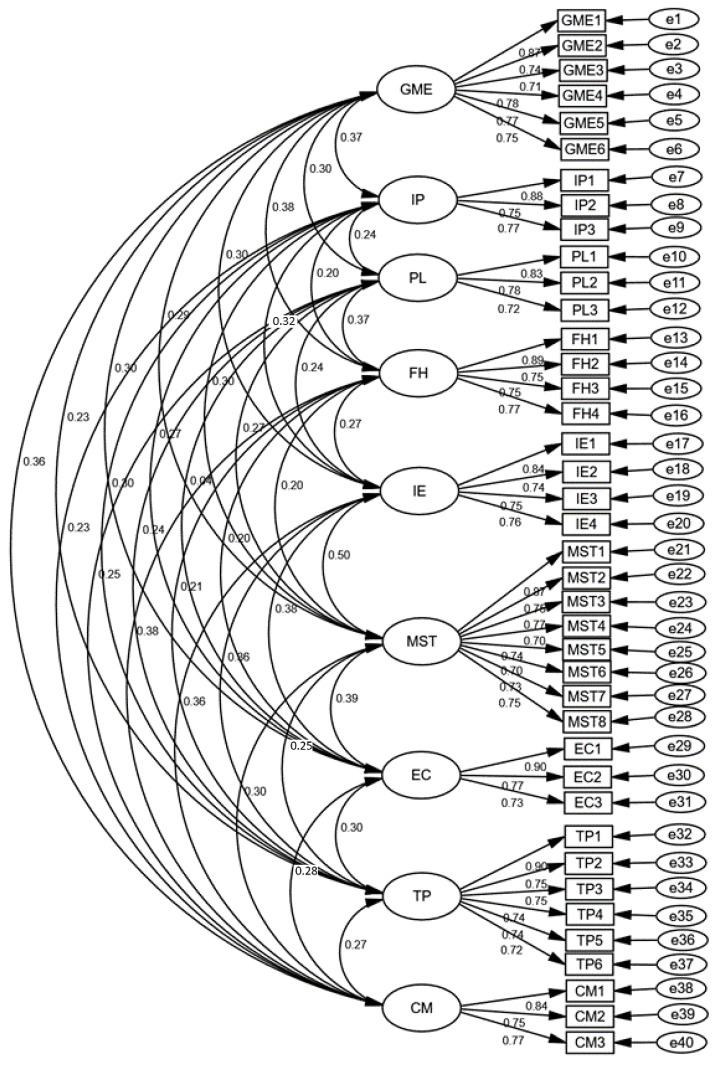
The 9-factor correlated model of EFL writing strategies for SRL with standardized regression weight and correlations (Model 1). Note. TP = text processing; CM = course memory; IP = ideal planning; GME = goal-oriented monitoring and evaluating; PL = peer learning; FH = feedback handling; IE = interest enhancement.

Construct validity examines the consistency between the experimental results and the theoretical framework. Data were subjected to three hypothesized models to validate the multidimensional model as well as WSSRLQ. Figure 1 shows the results of the 9-factor correlated model of EFL writing strategies for SRL, including text processing, course memory, idea planning, goal-oriented monitoring and evaluation, peer learning, feedback handling, interest enhancement, motivational self-talk, and emotional control (Model 1). The loadings of these nine factors ranged from 0.70 to 0.90. The goodness-of-fit indices were employed to assess the model-to-data fit, as presented in Table 4. This shows that all the goodness-of-fit indices are within the acceptable range. Thus, Model 1 exhibited acceptable validity in the EFL advanced learners’ group.

**Figure 2:**
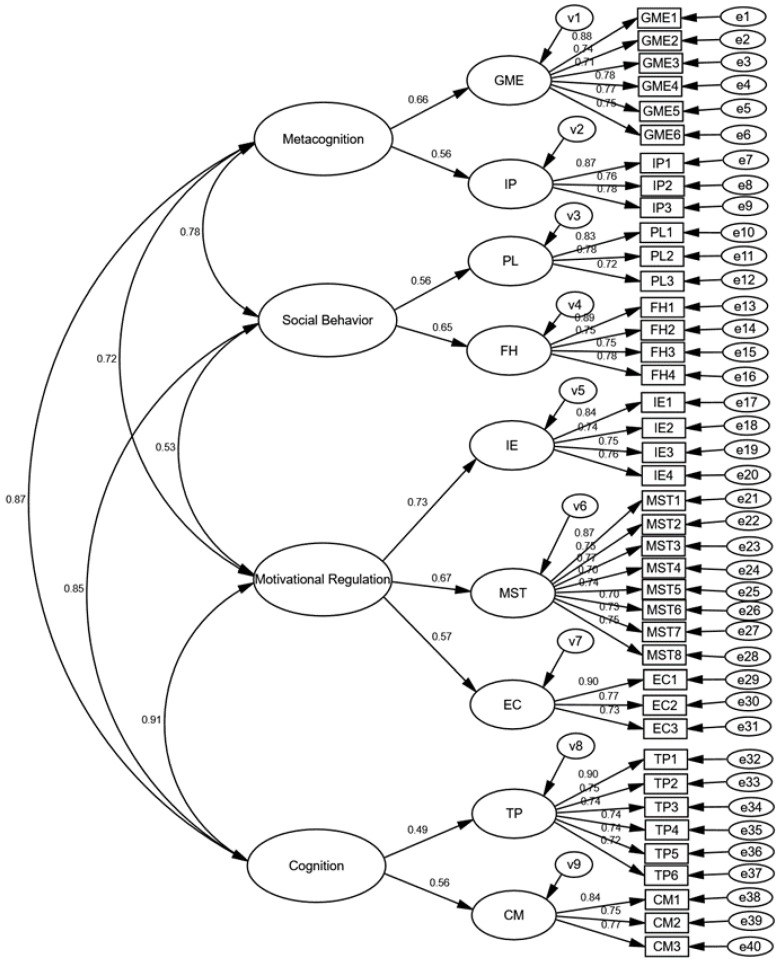
The 4-factor second-order model of EFL writing strategies for SRL (Model 2). Note. TP = text processing; CM = course memory; IP = ideal planning; GME = goal-oriented monitoring and evaluating; PL = peer learning; FH = feedback handling; IE = interest enhancement; MST = motivational self-talk; EC = emotional control.

Figure 2 shows the results of the four-factor second-order model of EFL writing strategies for SRL: the first order included text processing, course memory, idea planning, goal-oriented monitoring and evaluation, peer learning, feedback handling, interest enhancement, motivational self-talk, and emotional control; the second order included cognition, metacognition, social behavior, and motivational regulation (Model 2). The loadings of these nine factors ranged from 0.70 to 0.90; the loadings of these four factors ranged from 0.53 to 0.91. The goodness-of-fit indices were employed to assess the model-to-data fit, as presented in Table 4. This shows that all the goodness-of-fit indices were within the acceptable range. Thus, Model 2 exhibited acceptable validity in the EFL advanced learners’ group.

**Figure 3:**
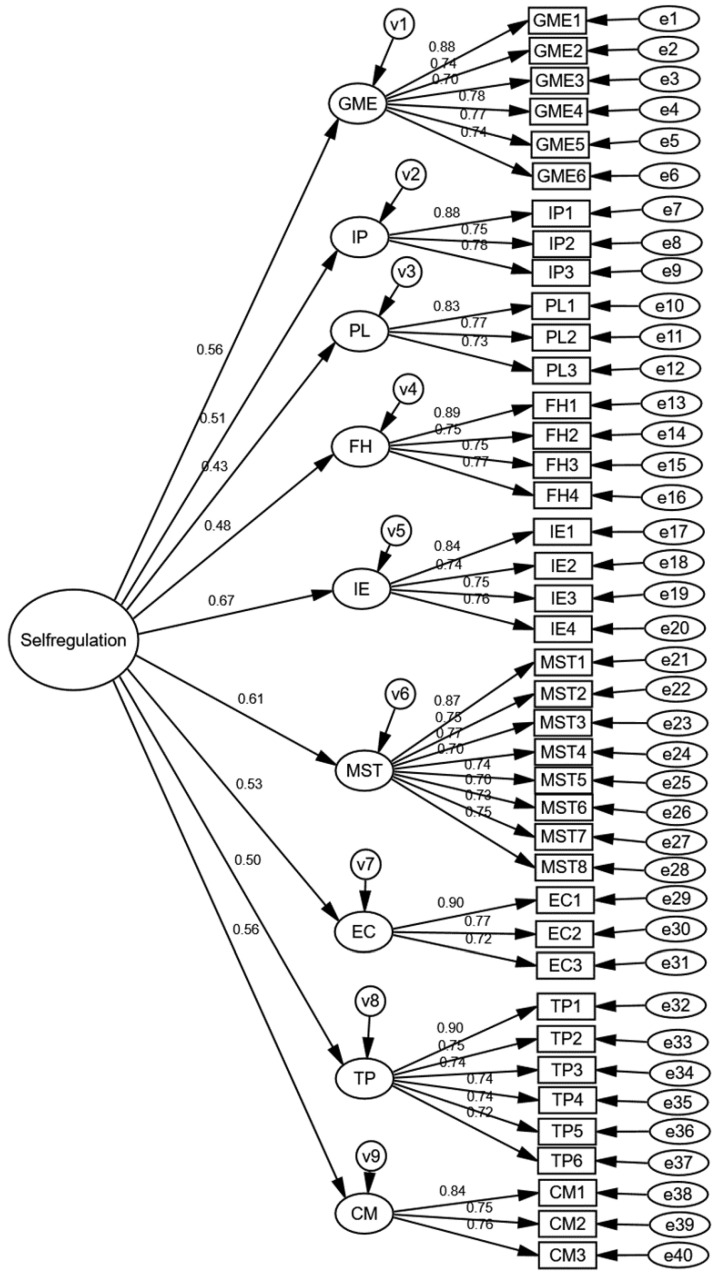
A one-factor second-order model of EFL writing strategies for SRL (Model 3). Note. TP = text processing; CM = course memory; IP = ideal planning; GME = goal-oriented monitoring and evaluating; PL = peer learning; FH = feedback handling; IE = interest enhancement; MST = motivational self-talk; EC = emotional control.

Figure 3 shows the results of the one-factor second-order model of EFL writing strategies for SRL: the first order included text processing, course memory, idea planning, goal-oriented monitoring and evaluation, peer learning, feedback handling, interest enhancement, motivational self-talk, and emotional control; the second order included self-regulation (Model 3). The loadings of these nine factors ranged from 0.70 to 0.90. The goodness-of-fit indices were employed to assess the model-to-data fit, as presented in Table 4. This shows that all the goodness-of-fit indices were within the acceptable range. Thus, Model 3 exhibited acceptable validity in the EFL advanced learners’ group.

Table 5 presents comparisons between the models. Fitness indices were selected from the absolute fitness index (χ^2^, RMSEA, ECVI), the valued-added fitness index (IFI, CFI, TLI), and the comprehensive fitness index (χ^2^/df, PGFI, PNFI, AIC) to provide a thorough view for the construct validity of the three models. The results show that all the models had a favorable model fit, confirming the multidimensional model of self-regulation as a hierarchical construct, with nine correlated writing strategies for SRL included in the four dimensions of cognition, metacognition, social behavior, and motivational regulation.

There was no significant improvement between Model 1 and Model 2 (x^2^_M2_ − x^2^_M1_ = 26.97; df_M2_ − df_M1_ = 21; *p* = 0.17). However, the indices of Model 2 (x^2^_M3_ − x^2^_M2_ = 30.88; df_M3_ − df_M2_ = 6; *p* < 0.00) and Model 1 (x^2^_M3_ − x^2^_M1_ = 57.86; df_M1_ − df_M3_ = 27; *p* < 0.00) significantly improved in fit over Model 3 (one high order factor model). The significant difference between the chi-square values suggests that Model 1 and Model 2 were significantly better than Model 3, with the four-factor second-order model as a hierarchical construct explaining the nine SRL strategies as the model of best fit in this study.

## 5. Discussion

This study aimed at validating self-regulated writing strategies for advanced EFL learners in China through a structural equation modeling analysis. Model 1 (nine-factor correlated model of EFL writing strategies for SRL), Model 2 (four-factor second-order model of EFL writing strategies for SRL) and Model 3 (one-factor second-order model of EFL writing strategies for SRL) all had acceptable model-fit indices. This means that the nine factors of self-writing strategies were conceptualized into four dimensions, namely cognition, metacognition, social behavior, and motivational regulation. All nine strategies can be accounted for in an integrated construct, namely self-regulation. In short, a hierarchic model with the higher order of self-regulation and the second order of nine self-regulated writing strategies belonging to four dimensions has been validated through the data from the samples in this study. These findings provide additional evidence of the cross-sample stability and the overall structural validity of the WSSRLQ, especially for advanced EFL learners’ self-regulation writing strategies. These findings also support the theoretical framework and the factor interaction of self-regulation learning in writing strategies in the literature [20,21,22,23,28].

In terms of model comparisons, this study found that all three models had acceptable model-fit indices and could explain constructs related to advanced EFL learners’ self-regulation writing strategies, but the indices in Model 1 (nine-factor correlated model of EFL writing strategies for SRL) and Model 2 (four-factor second-order model of EFL writing strategies for SRL) significantly improved on the indices in Model 3 (one-factor second-order model of EFL writing strategies for SRL). This means that the relevance of strategies and dimensions demonstrated a relatively better explanation than the convergence of strategies and dimensions for advanced EFL learners’ self-regulated writing strategies. This finding differed to some extent from those in previous studies, which showed that Model 3 had the best model fit. This difference may be due to the group of learners under investigation. Previous studies validated and compared models mainly in terms of educational level, including in EFL secondary schools [23,28] and universities [20,22]. This study investigated models on the basis of language proficiency level. Advanced learners are an especially notable group thanks to their frequency, flexibility, and control and thanks to the trends of their metacognitive strategy usage [29,30]. This difference in the findings highlights the importance of considering language proficiency level when investigating the validity of the WSSRLQ and other relevant models. Furthermore, these findings also add more evidence to research on self-regulated learning from the social cognitive perspective of the self-regulated learning process as an interplay of cognitive, metacognitive, behavioral, and environmental factors [89].

In terms of correlations between nine self-regulated writing strategies, the study found significant correlations (*p* < 0.05) between all strategies except emotional control and peer learning. Motivational self-talk and interest enhancement showed the highest correlation, 0.501, while motivational self-talk and feedback handling as well as idea planning and feedback handling showed the lowest correlation, 0.199. Regarding different dimensions, goal-oriented monitoring and evaluating in metacognition and feedback handling in social behavior showed the highest correlation, 0.385, while emotional control in motivational regulation and peer learning in social behavior showed the lowest correlation. This was not consistent with the studies by Teng and Zhang [20] or Teng and Huang [23]. Teng and Zhang focused on university EFL learners [20], and their study showed that goal-oriented monitoring and evaluating strategies in the metacognitive component and interest enhancement in motivational regulation had the highest correlation, 0.52, while goal-oriented monitoring and evaluating in metacognition and feedback handling in social behavior had the lowest correlation, 0.13. In Teng and Huang’s study focusing on secondary-school EFL learners [23], goal-oriented monitoring and evaluating strategies in the metacognitive dimension and motivational self-talk in motivational regulation showed the highest correlation, 0.535, while peer learning and feedback handling in social behavior showed the lowest correlation, 0.112.

The differences between studies indicate that advanced EFL learners display varying characteristics in self-regulation. Where general EFL learners whose goal-oriented monitoring and evaluating strategies were highly correlated with motivational regulation dimensions, advanced EFL learners showed a high correlation between these same strategies and feedback handling. This indicates that advanced learners are better equipped to incorporate feedback into their L2 writing process and adjust their goals accordingly. This further suggests that at this advanced stage, learners have already developed a high level of self-regulation and are able to use feedback to improve their writing skills. They may be more focused on methods for improving the quality of their writing, rather than simply being motivated to learn and use the English language. This is an important shift that occurs as learners progress and become increasingly focused on the actual process of language learning and the mastery of the language itself. Such individual variations should be considered when conducting relevant studies [24,58,59].

## 6. Conclusions

Using a structural equation modeling analysis, this study explored the validation of self-regulated writing strategies for advanced EFL learners. It has several implications for L2 writing settings. First, this study highlights the importance of considering individual differences, especially in language proficiency level, when researching self-regulated writing in an EFL context. Second, model comparisons on the WSSRLQ suggest that while model validity has been widely explored, model fit might vary across contexts and groups. Validity testing, judgment, and justification are complex and should be explained according to context and learner features. Third, the findings have implications for writing pedagogy. When L2 teachers teach writing strategies to EFL learners, it is necessary to learn about the target groups, especially their language proficiency levels. It would be useful to conduct diagnostic tests or needs analyses before teaching delivery.

Despite its contributions, this study inevitably has limitations. First, WSSRLQ was the only tool utilized to study participants’ use of self-regulated writing strategies. The strategies self-reported by participants may not include all the strategies they use in reality, as they may have failed to recall some strategies when completing the questionnaire. Second, the subjects were selected among Chinese undergraduates; therefore, the sample is relatively small. Moreover, individual differences (e.g., learners’ genders and academic majors) were not considered. However, according to some research, women performed better at writing than men; students majoring in humanities performed better at writing than those in natural sciences and engineering majors [90]. Third, CET 6 (a national English test administered by the National Education Examinations Authority of China and among the most widely used English tests in China) was employed to assess EFL learners’ proficiency levels. However, while this test is currently mapped to the CEFR, some university students majoring in English were not included in this study, because they take a professional-level English test (TEM-8) instead, and it is currently impossible to map their performance to specific CEFR levels. This is because the official mapping of TEM-8 scores to CEFR levels has not yet been released.

Given the limitations of this study, several suggestions for further research can be proposed. First, other methods of data collection (reading aloud, interviews, and reflection journals) are recommended for use in future studies to obtain longitudinal and dynamic data on EFL learners’ self-regulated writing strategies. Second, more participants from EFL contexts should be recruited by future researchers to add to the existing findings, especially advanced learners assessed by using tests other than CET 6. Third, multiple individual variances should be considered simultaneously in empirical studies to investigate the interactions between and the effects of these factors (e.g., gender, academic major, educational background, and language proficiency level). Fourth, in this study, participants’ CET 6 writing scores were obtained at different times. In future studies, if conditions permit, learners’ writing scores should be obtained from the same test at the same time. Lastly, future studies could validate the questionnaire or model not only from test takers’ perspectives but also from learners’ and teachers’ perspectives. For example, future research could explore the possibility of utilizing the questionnaire to improve writing and to give teachers feedback on students’ preferences for writing strategies, which could allow them to offer tailored classroom guidance.

## Figures and Tables

**Table 1:** Summary of the WSSRLQ instrument.

Strategy Domain		Questionnaire Items
Cognition	Text Processing (TP)	When writing, I use some literary devices to make the composition more interesting.
		When writing, I check grammar mistakes.
		When writing, I check spelling and punctuation.
		When writing, I check the structure for logical coherence.
		When writing, I check the cohesiveness or connection among sentences.
		When writing, I check whether the topic and the content have been clearly expressed.
	Course Memory (CM)	I write useful words and expressions taught in writing courses to help me remember them.
		I speak out useful words and expressions taught in writing courses to help me remember them.
		I read my class notes and the course material over and over again to help me remember them.
Metacognition	Idea Planning (IP)	Before writing, I read related articles to help me plan.
		Before writing, I use the internet to search for related information to help me plan.
		Before writing, I think about the core elements of good composition that I have learned to help me plan.
	Goal-Oriented Monitoring and Evaluating (GME)	When learning to write, I set up goals for myself in order to direct my learning activities.
		When learning to write, I check my progress to make sure I achieve my goal.
		I evaluate my mastery of the knowledge and skills learned in writing courses.
		I monitor my learning process in writing courses.
		When writing, I tell myself to follow my plan.
		When learning to write, I set up a learning goal to improve my writing.
Social Behavior	Peer Learning (PL)	I brainstorm with my peers to help me write.
		I discuss with my peers to gain more ideas to write with.
		I work with my peers to complete a writing task.
	Feedback Handling (FH)	I am open to peer feedback on my writing.
		I am open to teacher feedback on my writing.
		I try to improve my English writing on the basis of peer feedback.
		I try to improve my English writing on the basis of teacher feedback.
Motivational Regulation	Interest Enhancement (IE)	I look for ways to bring more fun to the learning of writing.
		I choose interesting topics to practice writing.
		I connect the writing task with my real life to intrigue me.
		I try to connect the writing task with my personal interests.
	Motivational Self-Talk (MST)	I remind myself about how important it is to earn good grades in writing courses.
		I tell myself that it is important to practice writing to outperform my peers.
		I compete with other students and challenge myself to do better than them in writing courses.
		I tell myself to practice writing to earn good grades.
		I tell myself that I need to keep studying to improve my writing competence.
		I persuade myself to work hard in writing courses to improve my writing skills and knowledge.
		I persuade myself to keep on learning in writing courses to find out how much I can learn.
		I tell myself that I should keep on learning in writing courses to become good at writing.
	Emotional Control (EC)	I tell myself not to worry when taking a writing test or answering questions in writing courses.
		I tell myself to keep on writing when I want to give it up.
		I find ways to regulate my mood when I want to give up on writing.

Note. All items used a 7-point response scale (i.e., 1 = strongly disagree; 7 = strongly agree).

**Table 2:** The reliability analysis of the WSSRLQ questionnaire.

Questionnaire and Its Dimensions	Number of Questions	Cronbach’s Coefficient	CR
Text Processing (TP)	6	0.901	0.906
Course Memory (CM)	3	0.797	0.823
Idea Planning (IP)	3	0.851	0.864
Goal-Oriented Monitoring and Evaluating (GME)	6	0.897	0.902
Peer Learning (PL)	3	0.809	0.826
Feedback Handling (FH)	4	0.865	0.874
Interest Enhancement (IE)	4	0.844	0.855
Motivational Self-Talk (MST)	8	0.906	0.910
Emotional Control (EC)	3	0.822	0.848

**Table 3:** Convergent validity of the 9-factor correlated model.

Construct	Item	Estimate (Standardized)	S.E.	*p*-Value	CR	AVE
GME	GME1	0.878			0.897	0.595
	GME2	0.74	0.044	***		
	GME3	0.704	0.046	***		
	GME4	0.784	0.044	***		
	GME5	0.766	0.044	***		
	GME6	0.744	0.044	***		
IP	IP1	0.876			0.844	0.645
	IP2	0.751	0.05	***		
	IP3	0.777	0.051	***		
PL	PL1	0.833			0.82	0.604
	PL2	0.77	0.064	***		
	PL3	0.725	0.059	***		
FH	FH1	0.89			0.858	0.602
	FH2	0.746	0.048	***		
	FH3	0.751	0.046	***		
	FH4	0.771	0.046	***		
IE	IE1	0.84			0.855	0.597
	IE2	0.736	0.052	***		
	IE3	0.753	0.051	***		
	IE4	0.757	0.052	***		
MST	MST1	0.873			0.912	0.565
	MST2	0.745	0.043	***		
	MST3	0.766	0.043	***		
	MST4	0.702	0.046	***		
	MST5	0.736	0.046	***		
	MST6	0.701	0.044	***		
	MST7	0.727	0.044	***		
	MST8	0.748	0.045	***		
EC	EC1	0.901			0.843	0.643
	EC2	0.77	0.048	***		
	EC3	0.725	0.047	***		
TP	TP1	0.895			0.896	0.59
	TP2	0.753	0.044	***		
	TP3	0.745	0.044	***		
	TP4	0.736	0.045	***		
	TP5	0.742	0.044	***		
	TP6	0.723	0.043	***		
CM	CM1	0.84			0.83	0.62
	CM2	0.754	0.055	***		
	CM3	0.765	0.052	***		

Note. *** *p* < 0.01.

**Table 4:** Discriminant validity and intercorrelations of the 9-factor correlated model.

	CM	TP	EC	MST	IE	FH	PL	IP	GME
CM	1								
TP	0.274 **	1							
EC	0.283 **	0.304 **	1						
MST	0.303 **	0.289 **	0.393 **	1					
IE	0.364 **	0.361 **	0.385 **	0.501 **	1				
FH	0.376 **	0.207 *	0.203 *	0.199 *	0.271 **	1			
PL	0.247 **	0.244 **	0.043	0.265 **	0.244 **	0.366 **	1		
IP	0.234 **	0.301 **	0.272 **	0.302 **	0.323 **	0.199 *	0.24 **	1	
GME	0.356 **	0.227 **	0.297 **	0.289 **	0.301 **	0.385 **	0.295 **	0.367 **	1
AVE	0.595	0.645	0.604	0.602	0.597	0.565	0.643	0.59	0.62
$\sqrt{\mathrm{AVE}}$	0.771	0.803	0.777	0.776	0.773	0.752	0.802	0.768	0.787

Note. * *p* < 0.05, ** *p* < 0.01.

**Table 5:** Goodness-of-fit indices for competing models.

		Model 1	Model 2	Model 3
χ^2^		973.980	1000.952	1031.836
df		704	725	731
χ^2^/df	1 < χ^2^/df < 3	1.383	1.381	1.412
PGFI	>0.50	0.745	0.764	0.767
IFI	>0.90	0.958	0.957	0.953
TLI	>0.90	0.953	0.954	0.950
CFI	>0.90	0.958	0.957	0.953
PNFI	>0.50	0.780	0.800	0.802
RMSEA	<0.05	0.036	0.036	0.037
AIC		1205.980	1190.952	1209.836
ECVI		3.993	3.944	4.006

## Data Availability

The data of the work can be provided by the corresponding author upon request.

## References

[1] Cao Z., Yu S., Huang J. (2019). A qualitative inquiry into undergraduates’ learning from giving and receiving peer feedback in L2 writing: Insights from a case study. Stud. Educ. Eval..

[2] Su H., Lu X. (2022). Assessing pragmatic performance in advanced L2 academic writing through the lens of local grammars: A case study of ‘exemplification’. Assess. Writ..

[3] Sun Y.C., Lan G. (2023). A bibliometric analysis on L2 writing in the first 20 years of the 21st century: Research impacts and research trends. J. Second Lang. Writ..

[4] Yang Y.L., Sun Y., Chang P.Y., Li Y.M. (2019). Exploring the relationship between language aptitude, vocabulary size, and EFL graduate students’ L2 writing performance. TESOL Q..

[5] Yu S., Lee I. (2016). Exploring Chinese students’ strategy use in a cooperative peer feedback writing group. System.

[6] Zhang X., Lu X., Li W. (2022). Beyond differences: Assessing effects of shared linguistic features on L2 writing quality of two genres. Appl. Lingusit..

[7] Harris K.R., Graham S., MacArthur C.A., Reid R., Mason L.H., Zimmerman B.J., Schunk D.H. (2011). Self-regulated learning processes and children’s writing. Handbook of Self-Regulation of Learning and Performance.

[8] Deng X., Wang C., Xu J. (2022). Self-regulated learning strategies of Macau English as a foreign language learners: Validity of responses and academic achievements. Front. Psychol..

[9] López-Serrano S., Roca de Larios J., Manchón R.M. (2019). Language reflection fostered by individual L2 writing tasks: Developing a theoretically motivated and empirically based coding system. Stud. Second Lang. Acquis..

[10] Sun Q., Zhang L.J., Carter S. (2021). Investigating students’ metacognitive experiences: Insights from the English as a foreign language learners’ writing metacognitive experiences questionnaire. Front. Psychol..

[11] Zimmermann R. (2000). L2 writing: Subprocesses, a model of formulating and empirical findings. Learn. Instr..

[12] Zimmerman B.J., Zimmerman B.J., Schunk D.H. (2011). Motivational sources and outcomes of self-regulated learning and performance. Handbook of Self-Regulation of Learning and Performance.

[13] Schunk D.H., Greene J.A. (2018). Handbook of Self-Regulation of Learning and Performance.

[14] Rose H., Briggs J.G., Boggs J.A., Sergio L., Ivanova-Slavianskaia N. (2018). A systematic review of language learner strategy research in the face of self-regulation. System.

[15] Teng L.S., Zhang L.J. (2022). Can self-regulation be transferred to second/foreign language learning and teaching? Current status, controversies, and future directions. Appl. Lingusit..

[16] Tseng W.T., Dornyei Z., Schmitt N. (2006). A new approach to assessing strategic learning: The case of self-regulation in vocabulary acquisition. Appl. Lingusit..

[17] Finkbeiner C., Knierim M., Smasal M., Ludwig P.H. (2012). Self-regulated cooperative EFL reading tasks: Students’ strategy use and teachers’ support. Lang. Aware..

[18] Huang S.C. (2011). Convergent vs. divergent assessment: Impact on college EFL students’ motivation and self-regulated learning strategies. Lang. Test..

[19] Byrnes H., Byrnes H., Manchón R.M. (2014). Theorizing language development at the intersection of ‘task’ and L2 writing: Reconsidering complexity. Task-based Language Learning: Insights from and for L2 Writing.

[20] Teng L.S., Zhang L.J. (2016). A questionnaire-based validation of multidimensional models of self-regulated learning strategies. Mod. Lang. J..

[21] Teng L.S., Zhang L.J. (2018). Effects of motivational regulation strategies on writing performance: A mediation model of self-regulated learning of writing in English as a second/foreign language. Metacogn. Learn..

[22] Zhou S.A., Hiver P. (2022). The effect of self-regulated writing strategies on students’ L2 writing engagement and disengagement behaviours. System.

[23] Teng F., Huang J. (2019). Predictive effects of writing strategies for self-regulated learning on secondary school learners’ EFL writing proficiency. TESOL Q..

[24] Shen B., Bai B. (2022). Chinese university students’ self-regulated writing strategy use and EFL writing performance: Influences of self-efficacy, gender, and major. Appl. Linguist. Rev..

[25] Teng L.S., Yuan R.E., Sun P.P. (2020). A mixed-methods approach to investigating motivational regulation strategies and writing proficiency in English as a foreign language contexts. System.

[26] Chen J., Zhang L.J., Chen X.T. (2022). L2 learners’ self-regulated learning strategies and self-efficacy for writing achievement: A latent profile analysis. Lang. Teach. Res..

[27] Qin L., Jun Zhang L. (2019). English as a foreign language writers’ metacognitive strategy knowledge of writing and their writing performance in multimedia environments. J. Writ. Res..

[28] Teng M.F., Wang C., Zhang L.J. (2022). Assessing self-regulatory writing strategies and their predictive effects on young EFL learners’ writing performance. Assess. Writ..

[29] Kung F.W. (2019). Teaching second language reading comprehension: The effects of classroom materials and reading strategy use. Innov. Lang. Learn. Teach..

[30] Rose H., Harbon L. (2013). Self-regulation in second language learning: An investigation of the Kanji-learning task. Foreign Lang. Ann..

[31] Zimmerman B.J., Risemberg R. (1997). Becoming a self-regulated writer: A social cognitive perspective. Contemp. Educ. Psychol..

[32] Bailey D.R. (2019). Conceptualization of second language writing strategies and their relation to student characteristics. J. Asia TEFL.

[33] Machili I., Papadopoulou I., Kantaridou Z. (2020). Effect of strategy instruction on EFL students’ video-mediated integrated writing performance. J. Second Lang. Writ..

[34] Manchón R.M. (2018). Past and future research agendas on writing strategies: Conceptualizations, inquiry methods, and research findings. Stud. Second Lang. Learn. Teach..

[35] Griffiths C. (2020). Language learning strategies: Is the baby still in the bathwater?. Appl. Lingusit..

[36] Schneider J. (2022). Writing strategies as acts of identity. TESOL Q..

[37] Guo X., Huang L.S. (2020). Are L1 and L2 strategies transferable? An exploration of the L1 and L2 writing strategies of Chinese graduate students. Lang. Learn. J..

[38] Abbuhl R. (2012). Using self-referential pronouns in writing: The effect of explicit instruction on L2 writers at two levels of proficiency. Lang. Teach. Res..

[39] Vergaro C. (2004). Discourse strategies of Italian and English sales promotion letters. English Specif. Purp..

[40] Alastrué R.P., Plo Alastrué R., Pérez-Llantada C. (2015). English as a Scientific and Research Language: Debates and Discourses.

[41] Manchón R.M., Bitchener J., Stroch N., Wette R. (2017). The multifaceted and situated nature of the interaction between language and writing in academic settings. Advancing research agendas. Teaching Writing for Academic Purposes to Multilingual Students: Instructional Approaches.

[42] Mumin Z., Palfreyman D.M., van der Walt C. (2017). Academic Biliteracies: Multilingual Repertoires in Higher Education.

[43] Cumming A., Cumming A. (2006). Goals for ESL Writing Improvement in ESL and University Courses.

[44] Roca de Larios J., Coyle Y., Nicolás-Conesa F., Manchón R.M., Matsuda P.K. (2016). Focus on writers: Processes and strategies. The Handbook of Second and Foreign Language Writing.

[45] Sasaki M. (2007). Effects of study-abroad experiences on EFL writers: A multiple-data analysis. Mod. Lang. J..

[46] Cameron L.D., Jago L., Gellman M.D., Turner J.R. (2012). Cognitive strategies. Encyclopaedia of Behavioural Medicine.

[47] Manchón R.M., Grosjean F., Ping L. (2013). Writing. The Psycholinguistics of Bilingualism.

[48] Zhang Y.H., Xi J. (2023). Fostering self-regulated young writers: Dynamic assessment of metacognitive competence in secondary school EFL class. Lang. Assess. Q..

[49] Fadlelmula F.K., Cakiroglu E., Sungur S. (2015). Developing a structural model on the relationship among motivational beliefs, self-regulated learning strategies, and achievement in mathematics. Int. J. Sci. Math. Educ..

[50] Zimmerman B.J., Schunk D.H., DiBenedetto M.K., Guay F., Marsh H., McInerney D.M., Craven R.G. (2015). A personal agency view of self-regulated learning: The role of goal setting. Self-Concept, Motivation and Identity: Underpinning Success with Research and Practice.

[51] Pintrich P.R., Boekaerts M., Pintrich P.R., Zeidner M. (2000). The role of goal orientation in self-regulated learning. Handbook of Self-Regulation.

[52] Zimmerman B.J., Boekaerts M., Pintrich P.R., Zeidner M. (2000). Attaining self-regulation: A social cognitive perspective. Handbook of Self-Regulation.

[53] Zimmerman B.J. (2008). Investigating self-regulation and motivation: Historical background, methodological developments, and future prospects. Am. Educ. Res. J..

[54] Jansen R.S., van Leeuwen A., Janssen J., Conijn R., Kester L. (2020). Supporting learners’ self-regulated learning in massive open online courses. Comput. Educ..

[55] Hu J., Gao X. (2020). Appropriation of resources by bilingual students for self-regulated learning of science. Int. J. Biling. Educ. Biling..

[56] Şahin Kızıl A., Savran Z. (2018). Assessing self-regulated learning: The case of vocabulary learning through information and communication technologies. Comput. Assist. Lang. Learn..

[57] Teng L.S., Sun P.P., Xu L. (2018). Conceptualizing writing self-efficacy in English as a foreign language contexts: Scale validation through structural equation modelling. TESOL Q..

[58] Bol L., Garner J.K. (2011). Challenges in supporting self-regulation in distance education environments. J. Comput. High. Educ..

[59] Schunk D.H., Ertmer P.A., Boekaerts M., Pintrich P.R., Zeidner M. (2000). Self-regulation and academic learning: Self-efficacy enhancing interventions. Handbook of Self-Regulation.

[60] Boekaerts M., Zimmerman B.J., Schunk D.H. (2011). Emotions, emotion regulation, and self-regulation of learning. Handbook of Self-Regulation of Learning and Performance.

[61] Dörnyei Z. (2005). The Psychology of the Language Learner: Individual Differences in Second Language Acquisition.

[62] Oxford R.L. (2017). Teaching and Researching Language Learning Strategies Self-Regulation in Context.

[63] Abdelhalim S.M. (2022). An investigation into English majors’ self-regulated writing strategies in an online learning context. Lang. Teach. Res..

[64] Teng L.S., Zhang L.J. (2020). Empowering learners in the second/foreign language classroom: Can self-regulated learning strategies-based writing instruction make a difference?. J. Second Lang. Writ..

[65] Zhang Y.N., Dong L.Q. (2022). A study of the impacts of motivational regulation and self-regulated second-language writing strategies on college students’ proximal and distal writing enjoyment and anxiety. Front. Psychol..

[66] Winne P.H., Zimmerman B.J., Schunk D.H. (2011). A cognitive and metacognitive analysis of self-regulated learning. Handbook of Self-Regulation of Learning and Performance.

[67] Umamah A., El Khoiri N., Widiati U., Wulyani A.N. (2022). EFL university students’ self-regulated writing strategies: The role of individual differences. J. Lang. Educ..

[68] Sun T., Wang C. (2020). College students’ writing self-efficacy and writing self-regulated learning strategies in learning English as a foreign language. System.

[69] Bai B., Guo W. (2018). Influences of Self-Regulated Learning Strategy Use on Self-Efficacy in Primary School Students’ English Writing in Hong Kong. Read. Writ. Q..

[70] Bai B., Guo W. (2019). Motivation and self-regulated strategy use: Relationships to primary school students’ English writing in Hong Kong. Lang. Teach. Res..

[71] Mallahi O. (2020). Examining the extent of self-regulatory strategy use and writing competence of Iranian EFL learners. Appl. Linguist. Res. J..

[72] Ma R., Oxford R.L. (2014). A diary study focusing on listening and speaking: The evolving interaction of learning styles and learning strategies in a motivated, advanced ESL learner. System.

[73] Ministry of Education of the People’s Republic of China (2018). Curriculum and Standards for Senior High School English Teaching.

[74] Ministry of Education of the People’s Republic of China (2015). Guidelines on College English Teaching.

[75] Xu J., Fan Y. (2017). The evolution of the college English curriculum in China (1985–2015): Changes, trends and conflicts. Lang. Policy.

[76] Zhang Q. (2022). Impacts of world Englishes on local standardized language proficiency testing in the expanding circle: A study on the College English Test (CET) in China. Engl. Today.

[77] Li H., He L.A. (2015). Comparison of EFL raters’ essay-rating processes across two types of rating scales. Lang. Assess. Q..

[78] Council of Europe (2020). Common European Framework of Reference for Languages: Learning, Teaching, Assessment–Companion Volume.

[79] Ministry of Education of the People’s Republic of China, National Language Commission of the People’s Republic of China (2018). China’s Standards of English Language Ability.

[80] Jin Y., Jie W., Wang W. (2022). Exploring the alignment between the College English Test and language standards. Foreign Lang. World.

[81] Amenos-Pons J., Ahern A., Guijarro-Fuentes P. (2019). Feature reassembly across closely related languages: L1 French vs. L1 Portuguese learning of L2 Spanish past tenses. Lang. Acquis..

[82] Lecouvet M. (2021). Non-canonical word order as a measure of syntactic complexity in advanced L2 German. IRAL Int. Rev. Appl. Linguist. Lang. Teach..

[83] Lundell F.F., Lindqvist C., Edmonds A. (2018). Productive collocation knowledge at advanced CEFR levels: Evidence from the development of a test for advanced L2 French. Can. Mod. Lang. Rev..

[84] Piniel K., Albert Á. (2018). Advanced learners’ foreign language-related emotions across the four skills. Stud. Second Lang. Learn. Teach..

[85] Field A.P. (2009). Discovering Statistics Using SPSS.

[86] Nunnally J.C., Bernstein I.H. (1994). Psychometric Theory.

[87] Hair J.F. (1998). Multivariate Data Analysis.

[88] Fornell C., Larcker D.F. (1981). Evaluating structural equation models with unobservable variables and measurement error. J Mark. Res..

[89] Zimmerman B.J. (2013). From cognitive modelling to self-regulation: A social cognitive career path. Educ. Psychol..

[90] Oppenheimer D., Zaromb F., Pomerantz J.R., Williams J.C., Park Y.S. (2017). Improvement of writing skills during college: A multi-year cross-sectional and longitudinal study of undergraduate writing performance. Assess. Writ..

